# EⅡB Mutation Reduces the Pathogenicity of *Listeria monocytogenes* by Negatively Regulating Biofilm Formation Ability, Infective Capacity, and Virulence Gene Expression

**DOI:** 10.3390/vetsci11070301

**Published:** 2024-07-02

**Authors:** Caixia Liu, Ruixuan Qian, Weidi Shi, Lijun Kou, Jing Wang, Xun Ma, Huijie Ren, Shengjie Gao, Jingjing Ren

**Affiliations:** College of Animal Science and Technology, Shihezi University, Shihezi 832000, China; liucaixia0402@163.com (C.L.); qianruixuan@stu.shzu.edu.cn (R.Q.); shiweidi1127@163.com (W.S.); klj175@163.com (L.K.); renhuijie@stu.shzu.edu.cn (H.R.); gaosj1025@163.com (S.G.); renjingjing0524@163.com (J.R.)

**Keywords:** LIPI-4, adhesion, invasion, intracellular proliferation, inflammatory cytokines

## Abstract

**Simple Summary:**

*Listeria* pathogenicity island 4 (LIPI-4)—which is composed of six genes, including EⅡB—has been identified as a specific virulence factor closely related to infections of the nervous system and the placenta. This study examined the virulence effect of LIPI-4 EⅡB. Deletion of EⅡB reduced the biofilm formation ability and cell intracellular proliferation capacity of *Listeria monocytogenes*, as well as bacterial load in mice. In addition, EⅡB was found to regulate the transcription levels of genes related to virulence and biofilm formation. These findings provide a foundation for further examinations of the pathogenic mechanisms of LIPI-4 and EⅡB in *L. monocytogenes*.

**Abstract:**

To explore the role of the membrane permease ⅡB (EⅡB) gene of *Listeria* pathogenicity island 4 (LIPI-4) in the virulence of *Listeria monocytogenes*, both an EⅡB deletion strain (∆EⅡB) and a complemented strain were constructed. In vitro experiments demonstrated that EⅡB deletion affected the biofilm formation ability of the wild-type strain (Lm928). Moreover, this deletion decreased the intracellular proliferation abilities of *L. monocytogenes*. Mice infected with ∆EⅡB survived longer and experienced less weight loss on days 1, 2, and 3 post-infection. The bacterial load in the liver tissue of ∆EⅡB-infected mice was significantly reduced, and a considerable decrease in the blood levels of inflammatory cytokines IL-β, IL-6, IL-10, and TNF-α were observed. Following EⅡB deletion, 65% (13/20) of genes were downregulated, 25% (5/20) were upregulated, and 10% (2/20) showed no change. These findings suggest that EⅡB deletion may reduce both the in vivo and in vitro virulence levels as well as the biofilm formation ability of Lm928 by downregulating the transcription levels of genes associated with virulence and biofilm formation. These findings provide a foundation for further examining the pathogenic mechanisms of LIPI-4 and EⅡB in *L. monocytogenes*.

## 1. Introduction

*Listeria monocytogenes* is a widely distributed, Gram-positive bacterium that can be found in various environments [1]. *L. monocytogenes* can survive inside cells and is transmitted via the ingestion of infected food. It is the causative agent of listeriosis, a severe zoonotic disease that affects both humans and animals. The capacity of *L. monocytogenes* to thrive in cold temperatures, create biofilms, and endure in food-processing facilities is a notable impediment to food safety [2]. In 2019, listeriosis had the highest case fatality rate (8.9%) among outbreak-related diseases in Europe [3]. In the USA, approximately 1600 listeriosis cases are reported annually, which result in ~260 deaths [4]. Shanxi Bethune Hospital in China found that the detection rate of *L. monocytogenes* increased between January 2017 and January 2023, and today, it is only outranked by *Streptococcus pneumoniae* [5]. *L. monocytogenes* can cross the intestinal barrier via intestinal epithelial cells, from where it reaches the liver, spleen, lymphatic system, and blood circulatory system, where it multiplies. Finally, it spreads through the bloodstream to the brain and placenta, thereby causing symptoms such as meningitis, sepsis, abortion, and mononucleosis in both humans and animals [6].

*Listeria* pathogenicity island 4 (LIPI-4) was discovered only after multilocus sequence typing-based analysis of *L. monocytogenes* strains acquired from human listeriosis and contaminated food; this discovery showed that based on their presence in food, sequence type 4 (ST4) strains were more commonly linked to human diseases than expected, identifying ST4 as a highly virulent strains of *L. monocytogenes* [7]. Whole-genome sequencing analysis of ST4 showed that these strains possess a cluster of genes labeled as the phosphotransferase system (PTS) of the cellobiose family (maltose-6′-P-glucosidase, transcriptional antitermination, uncharacterized protein associated with PTS systems, and membrane permease EⅡA, EⅡB, and EⅡC), while other reference strains do not. Evaluation of virulence experiments in mice models identified a distinct virulence component linked to infections of the central nervous system and the placenta; this gene cluster was named LIPI-4 [7]. Although LIPI-4 was initially assumed to be specific to the highly virulent strain ST4, subsequent whole-genome sequencing analyses also identified the presence of LIPI-4 in several other strains. These include the emerging strain ST382, which has been associated with a recent outbreak of listeriosis in the USA linked to agricultural products [8]. It has been demonstrated that all ST87-type *L. monocytogenes* isolated in China contained LIPI-4 [9]. Remarkably, research showed that LIPI-4 is also present in *L. innocua*, and that intact LIPI-4 is stably conserved in all *L. innocua*; further, it is located at the same position as LIPI-4 in *L. monocytogenes* [10]. LIPI-4 is highly conserved in *L. innocua* and in various strains of *L. monocytogenes*. However, the similarity between LIPI-4 in *L. innocua* and LIPI-4 in *L. monocytogenes* is low (83.7–84.0%), suggesting that LIPI-4 in *L. innocua* and LIPI-4 in *L. monocytogenes* are two different variants [10].

LIPI-4 is essentially a phosphoenolpyruvate PTS. Kundig discovered PTS in 1964 and suggested that it may catalyze sugar transport and sugar phosphorylation [11]. The typical bacterial PTS is composed of the phosphoenolpyruvate-dependent protein kinase enzyme I (EI), the histidine-containing protein (HPr), and substrate-specific enzyme Ⅱ (EⅡ) complexes [12]. During bacterial transport, EI undergoes autophosphorylation by acquiring a phosphate group from phosphoenolpyruvate; this phosphate group is subsequently transferred to the histidine residue of Hpr. HPr then transfers the phosphate group sequentially to EⅡA, EⅡB, and EⅡC. At this stage, incoming extracellular carbohydrates receive the phosphate group from EⅡC, resulting in the formation of phosphorylated carbohydrates that enter the glycolytic pathway. EⅡC has the ability to identify sugar and facilitate its transport into cells. Therefore, EⅡC is responsible for determining the precise specificity of sugar transport in the PTS system [12]. PTS can not only sense and respond to external stimuli but also to the internal metabolic state of cells [13]. In addition to sugar uptake, certain PTSs are associated with growth rate, biofilm formation, expression of virulence genes, and virulence [14]. It has also been shown that EⅡB plays different roles in PTS of other strains [15,16,17,18].

Five of the fifty-four *L. monocytogenes* strains previously isolated in the laboratory carry the LIPI-4 gene, among which Lm928 (ST87 type) isolated from Xinjiang frozen chicken is the most virulent [19]. Since the discovery of LIPI-4 in 2016 [7], research on LIPI-4 has focused on epidemiologic examinations [9,10,20,21,22] but the pathogenic mechanisms of LIPI-4 and the six genes that make up LIPI-4 have not been examined. Therefore, the purposes of this study were to construct EⅡB deletion and complementary strains of Lm928 LIPI-4, as well as to explore whether EⅡB affects the growth, biofilm formation, virulence, and gene regulation of Lm928. This paper provides a foundation for further research towards understanding the role of LIPI-4 in *L. monocytogenes* based on the biological function of LIPI-4 EⅡB.

## 2. Materials and Methods

### 2.1. Bacterial Strains and Cell Lines

Lm928 was isolated and identified by the Analysis and Test Center, Xinjiang Academy of Agricultural Reclamation Sciences (Shihezi, China), and was cultured using brain heart infusion (BHI) (Hopebio, Qingdao, China). The temperature-sensitive shuttle vector pKSV7 was obtained from Zhejiang University, China, while the integration plasmid pIMK2 was kindly provided by Professor Yuelan Yin of Yangzhou University, China. The *Escherichia coli* DH5α strain was purchased from Transgen Biotech (Beijing, China), and was cultured using Luria–Bertani medium (Hopebio, Qingdao, China). Human choriocarcinoma cells JEG-3, immortalized human cerebrum microvascular endothelial cells (hCMEC/D3), and mouse intestinal epithelial cell line MODE-K were obtained from BeNa Culture Collection (Xinyang, China; https://www.bncc.com/ (accessed on 20 April 2024)). These cells were cultured in minimum essential medium (MEM) (Procell, Wuhan, China) and Dulbecco’s modified eagle medium (DMEM) (Gibco, Suzhou, China) with 10% fetal bovine serum (FBS) (Excell Bio, Shanghai, China) under 5% CO_2_. Human colon adenocarcinoma cells and mouse macrophages RAW264.7 were obtained from Procell (Wuhan, China; https://www.procell.com.cn/ (accessed on 20 April 2024)) and were cultured in MEM (20% FBS) and DMEM (10% FBS), respectively, under 5% CO_2_.

### 2.2. Animals

Six- to eight-week-old Kunming mice were purchased from the Animal Experimental Center of Xinjiang Medical University, China. Six- to eight-week-old C57BL/6 female specific pathogen free mice were purchased from Henan Skebeth Biotechnology (Anyang, China). Mice were housed under controlled humidity (40–70%) and temperature (21–26 °C) conditions throughout the experiment. All animal experimental protocols conformed to the rules of the National Guidelines for Housing and Care of Laboratory Animals (China). All animal experiments were approved by the Biology Ethics Committee of Shihezi University (Approval Number: A2021-26).

### 2.3. Experimental Design

**Figure 1:**
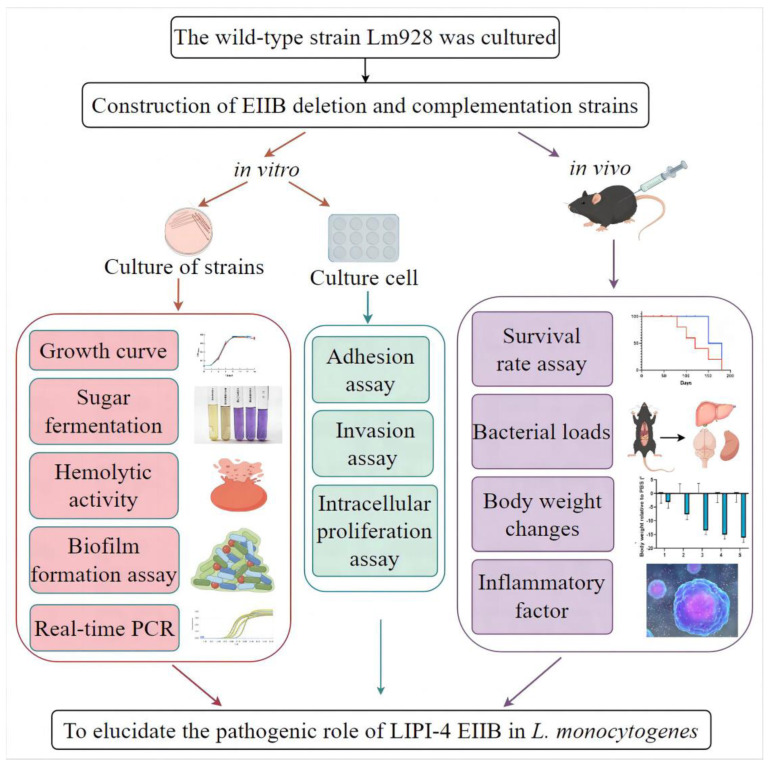
Experimental design.

The experimental design of the current study is described below and illustrated in Figure 1. First, the EⅡB deletion mutant (∆EⅡB) was constructed by homologous recombination. In the in vitro study, growth curve, sugar fermentation, biofilm formation capacity, hemolytic activity, and transcription levels of genes involved in virulence and biofilm formation were examined. To evaluate the role of ∆EⅡB on intracellular bacterial survival, adhesion, invasion, and intracellular proliferation assays in cell model were used, including hCMEC/D3, MODE-K, RAW264.7, and JEG-3. In the in vivo study, the virulence level was evaluated by mouse survival rate, bacterial burden in various tissues (liver, spleen, and brain), body weight change, and serum level of inflammatory factor.

### 2.4. Primers

Primers were designed using the *L. monocytogenes* strain Lm928 genome in GenBank (accession number NZ_CP046478.1) using Oligo 6.0 software (Supplementary Table S1). The upstream homology arm of the EⅡB gene was amplified using primers F1 and R1, while the downstream homology arm was amplified using primers F2 and R2. D-F and D-R primers were used to verify the deletion strain. Complementary strains were constructed with EⅡB gene amplification primers C-F and C-R.

### 2.5. Construction of the Mutant and Complemented Strain

Using the Lm928 genome as template, polymerase chain reaction (PCR) amplification of EⅡB gene upstream (F1 and R1 primers) and downstream (F2 and R2 primers) homologous arms were conducted. The mastermix composition consisted of a total of 20 μL, including Taq Plus Master Mix Ⅱ (Vazyme, Nanjing, China) 10 μL, forward primer 1 μL, reverse primer 1 μL, DNA 1 μL, and nuclease-free water 7 μL. The reaction program used denaturation at 95 °C for 5 min, followed by 35 cycles of denaturation at 95 °C for 30 s, annealing at 58 °C for 30 s, and extension at 72 °C for 30 s; final extension was conducted at 72 °C for 10 min. The PCR products were purified and recovered using the FastPure Gel DNA Extraction Mini Kit (Vazyme, Nanjing, China). Splicing with overlap extension PCR was used to fuse upstream and downstream arms, resulting in the ΔEⅡB fragment. The reaction system and reaction conditions are detailed in Supplementary Tables S2 and S3.

This ΔEⅡB fragment was then ligated to the pKSV7 fragment (chloramphenicol resistance) and sequenced (Sangon Biotech, Shanghai, China). The accurately sequenced pKSV7ΔEⅡB plasmid was electrotransferred into Lm928 cells. Then, homologous recombination was carried out in the presence of chloramphenicol (10 μg/mL) at 42 °C, and PCR amplification was performed using D-F and D-R primers. pKSV7 plasmids were eliminated under no-resistance conditions at 30 °C and mutant strains were tested for chloramphenicol sensitivity at intervals of five generations. The ΔEⅡB strain was then incubated at 37 °C under shaking for 25 generations to test its genetic stability, and PCR amplification was performed at five-generation intervals using the primers D-F and D-R. ΔEⅡB strain was identified by PCR amplification, sequencing, and real-time PCR (Supplementary Table S4).

Following amplification, the PCR product was digested by restriction enzymes *Pst*Ⅰ and *Xho*Ⅰ. The PCR product was then purified using a FastPure Gel DNA Extraction Mini Kit (Vazyme, Nanjing, China) (Vazyme, Nanjing, China). The purified PCR product was ligated into the *L. monocytogenes* site-specific integration vector pIMK2, which was digested with the same restriction enzymes; then, the recombinant plasmid pIMK2-EⅡB was constructed. For electroporation, 50 μL of electrocompetent cells were mixed with 10 μL of plasmid ligation mixture. The recombinant plasmid pIMK2-EⅡB was electrotransformed into ΔEⅡB, which was then cultured in BHI agar medium containing kanamycin (final concentration of 50 μg/mL) at 30 °C. CΔEⅡB was identified by PCR amplification, sequencing, and real-time PCR.

### 2.6. Analysis of Biological Characteristics of L. monocytogenes Strains

The growth curve was quantified as previously described [14]. *L. monocytogenes* strains were grown in BHI medium overnight at 37 °C, and the optical density at 600 nm (OD_600_) of the cultures was measured by enzyme labeled instrument (Power Wave Xs, BioTek, Vermont, USA). The OD_600_ of cultures was adjusted to 0.5 using BHI medium, washed twice with PBS, and then resuspended. The adjusted cultures were inoculated at 1:100 into fresh BHI medium or cellobiose as the sole carbon source medium (0.2% cellobiose, 1% tryptone, 0.3% beef extract powder, and 0.5% NaCl). Data for growth curves were collected by measuring changes in OD_600_ at 2 h intervals using a microplate reader over a total period of 14 h. Additionally, single colonies of Lm928, ΔEⅡB, and CΔEⅡB were selected and placed in fermentation tubes containing glucose, cellobiose, fructose, rhamnose, aesculin, and salicin (Hopebio, Qingdao, China). These tubes were then incubated at 37 °C for 24 h to assess sugar fermentation. The evaluation of the fermented sugar strain was based on the color of the fermentation tube, as specified in the provided instructions. The experiment was repeated three times.

### 2.7. Detection of Biofilm Formation

The biofilm formation ability of Lm928, ΔEⅡB, and CΔEⅡB was assessed following a previously described protocol [14] with minor modifications. Strains were recovered and single colonies were selected for cultivation in BHI medium at 37 °C under incubator shaking (IS-RDVI, Crystal, TA, USA) for 12–14 h. Subsequently, each well of a 96-well cell culture plate was filled with 150 μL BHI liquid medium, supplemented with 50 μL of bacterial culture solution at an OD_600_ value of approximately 0.2. After incubation at 37 °C for 8, 12, 24, 36, 48, and 72 h, the cultures within wells were discarded, followed by three washes using phosphate-buffered saline (PBS) to eliminate planktonic bacteria. Following the drying process, a staining step was performed by adding 100 μL of 0.1% crystal violet solution into each well and allowing it to stain for 30 min. After removal of the staining solution, the wells were washed five times using PBS. Crystal violet was dissolved in 95% ethanol for 20–30 min, and the relative biofilm formation was determined by measuring the OD_600_ value. The experiment was replicated thrice.

### 2.8. Determination of Infective Capacity of L. monocytogenes

To determine the infective capacity of *L. monocytogenes*, established methods [23] were used. The hCMEC/D3, MODE-K, RAW264.7, and JEG-3 cell lines were cultured in DMEM medium supplemented with 10% FBS and 1% penicillin–streptomycin solution (Solarbio, China). The hCMEC/D3, MODE-K, RAW264.7, and JEG-3 cells were seeded in 12-well plates and incubated at 37 °C under 5% CO_2_ atmosphere by an Incubator (Thermo 8000DH, Waltham, MA, USA) until a confluence of about 95% was reached. The numbers of cells were approximately 5 × 10^5^, 1.8 × 10^6^, 2 × 10^6^, and 9 × 10^5^ cells per well, respectively. Lm928, ΔEⅡB, and CΔEⅡB strains were cultured in BHI medium at 37 °C under constant agitation at a speed of 160 rpm/min for a duration of 12–14 h. Subsequently, hCMEC/D3, MODE-K, RAW264.7, and JEG-3 cells were infected with bacteria at multiplicity of infection values of 10, 10, 1, and 1, respectively. After 1 h of culture, cells were washed twice with PBS and digested using 0.25% trypsin (Solarbio, Beijing, China). To determine the rate of bacterial adhesion to cells, 0.2% Triton-X 100 (Biotopped, Beijing, China) was added to each well. Infected cells were further incubated in complete medium containing gentamicin (100 μg/mL) at 37 °C for 1 h, followed by washing twice with PBS, digesting with trypsin, and lysing using 0.2% Triton-X 100. This was conducted to determine the cell invasion rate of bacteria. Subsequently, infected cells were cultured at 37 °C for 1 h. After washing cells with PBS, complete medium containing gentamicin (10 μg/mL) was added to each well. Cells were collected as described above after 4, 8, and 12 h and bacterial proliferation inside the cells was determined. The collected samples were diluted using a 10-fold gradient dilution method [24] and were then used to coat a BHI agar plate. Colonies were counted after the culture plates were incubated for 24–36 h. The experiment was repeated three times.

### 2.9. Quantitation of Hemolytic Activity by Microplate Technique

The hemolytic activity was assayed as previously described, with the following minor modifications [25]: Healthy sheep blood was collected and defibrinated to prepare a 1% suspension of sheep red blood cells. Under aseptic conditions, the blood was transferred to 50 mL sterile centrifuge tubes and centrifuged (MuItifuge X1R, Thermo, Waltham, MA, USA) at 4 °C for 10 min at a speed of 2000 rpm. The supernatant and leukocyte layers were removed, and PBS was added to wash out broken red blood cells until the supernatant became clear after centrifugation. After discarding the supernatant, the required proportion of PBS was added to prepare a 1% suspension of sheep red blood cells in PBS for further use.

Lm928, ΔEⅡB, and CΔEⅡB strains were cultured in BHI medium for 12–14 h under constant shaking. The supernatant was adjusted using BHI medium to achieve an OD_600_ of 0.5, and the supernatant was then centrifuged (Micro17, Thermo, Waltham, MA, USA) at 8000 rpm for 5 min. Then, 100 μL of the supernatant from each strain after centrifugation was added to the V-well of a 96-well plate and diluted in PBS buffer by factors ranging from consecutive 2-fold dilutions. Uninoculated BHI medium served as negative control. An equal volume of prepared suspension containing 1% sheep red blood cells in PBS was added to each well, and the hemolytic activity of each strain was observed after incubation at 37 °C for 2 h. A total of 100 μL of the supernatant was aspirated to determine the OD_550_ value, and the experiment was replicated thrice.

### 2.10. Mouse Survival Rate Assay

The utilized methods have been described previously [23]. Three strains of *L. monocytogenes* were revived and incubated in BHI medium at 37 °C under constant shaking for 12–14 h. The bacterial precipitates were collected by centrifugation at 8000 rpm for 5 min, washed twice with PBS, resuspended, and adjusted to a concentration of approximately 10^8^ CFU/mL for further use. Female Kunming mice aged 6–8 weeks (35–45 g body weight) were randomly divided into PBS, Lm928, ΔEⅡB, and CΔEⅡB, groups, each consisting of six mice. The prepared bacterial suspension was intraperitoneally injected into mice (100 μL per mouse). Following this challenge, mortality of mice was observed continuously for seven days. Survival curve analysis and calculation of average survival times in mice were performed using GraphPad Prism 9.

### 2.11. Assessment of Bacterial Loads and Inflammatory Factor Levels

The experiment was carried out according to a previously described method [26]. *L. monocytogenes* wild-type Lm928, ΔEⅡB, and CΔEⅡB strains were grown overnight at 37 °C in BHI broth under constant shaking. The bacterial solution was centrifuged at 8000 rpm for 5 min, the medium was discarded, and cells were washed once with PBS. The bacterial solution concentration was adjusted to approximately 10^6^ CFU/mL. Female C57BL/6 mice aged 6–8 weeks (16–19 g body weight) were randomly divided into four groups, including PBS, Lm928, ΔEⅡB, and CΔEⅡB groups, with six mice per group. The mice in each group were intraperitoneally injected with 0.2 mL of the bacterial solution, and the body weight of mice was measured on days 0, 1, 2, and 3 after artificial infection. On the third day after artificial infection, mouse serum was collected, the liver, spleen, and brain tissues were removed under sterile conditions, and tissues were fully ground with PBS. After 10-fold gradient dilution, liver, spleen, and brain homogenates were plated onto BHI agar plates and incubated at 37 °C for 24–36 h.

The contents of Interleukin (IL)-1β, IL-6, IL-10, and tumor necrosis factor (TNF)-α in the serum of mice were measured by an enzyme-linked immunosorbent assay kit (Proteintech, Wuhan, China), strictly following the manufacturer’s instructions.

### 2.12. Real-Time PCR

Lm928, ΔEⅡB, and CΔEⅡB strains were cultured in BHI medium under shaking at 37 °C, for 14–16 h. The cells were then collected by centrifugation, washed with PBS, and flash-frozen in liquid nitrogen. Total RNA was extracted using an RNA extraction kit (TransGen Biotech, Beijing, China). The quality of RNA was tested through electrophoresis, followed by reverse transcription to obtain the corresponding cDNA, using a TransScript^®^ Uni All-in-One First-Strand cDNA Synthesis SuperMix for qPCR (One-Step gDNA Removal) (TransGen Biotech, Beijing, China). A control without reverse transcriptase was also used to determine whether the template for qPCR was derived from cDNA. These steps were strictly followed according to the manufacturer’s instructions. This cDNA was then used for the qRT-PCR reaction using PerfectStart^®^ Green qPCR SuperMix (TransGen Biotech, Beijing, China) according to the manufacturer’s protocol on the LightCycler 96 (Roche, Basel, Switzerland). Virulence and biofilm related gene transcription were tested, including *actA*, *agrA*, *agrB*, *agrC*, *degU*, *flaA*, *hly*, *inlA*, *inlB*, *inlC*, *inlP*, *lap*, *mpl*, *mogR*, *motA*, *motB*, *plcA*, *plcB*, *prfA*, and *sigB*. The mastermix composition consists of a total of 20 μL, including 2× PerfectStart Green qPCR SuperMix 10 μL, forward primer 0.4 μL, reverse primer 0.4 μL, cDNA 1 μL, and nuclease-free water 8.2 μL. The reaction program used denaturation at 95 °C for 30 s, followed by 45 cycles including denaturation at 94 °C for 5 s, annealing at 59 °C for 15 s, extension at 72 °C for 10 s; melting at 95 °C for 10 s, 65 °C for 60 s, and 97 °C for 1 s, followed by cooling at 37 °C for 30 s. The housekeeping gene *gyrB* was used as an internal control for normalization. After data collection, relative expression levels were calculated by the threshold cycle (2^−ΔΔCT^) method [26]. The primer sequences of target virulence genes are provided in Supplementary Table S4. The experiment was repeated three times.

### 2.13. Statistical Analysis

Statistical analysis was conducted using SPSS 20.0 software. A significance level of 0.05 was assumed to indicate statistical significance. One-way analysis of variance was employed for the analysis.

## 3. Results

### 3.1. Construction of EⅡB Deletion Strain and Complement Strain

**Figure 2:**
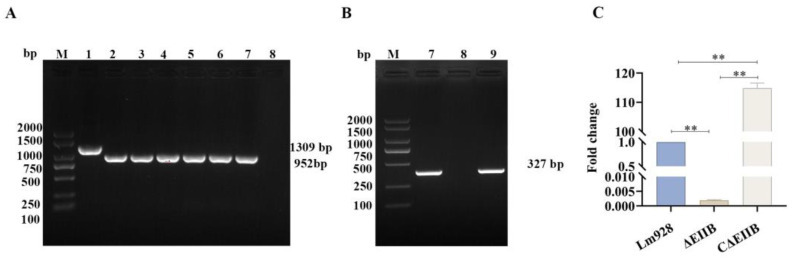
The EⅡB deletion and complement strains were successfully constructed. (**A**) Identification of the deletion strain and simultaneous detection of ∆EⅡB genetic stability using EⅡB-specific primers DF and DR. (**B**) Validation of EⅡB expression in the complemented strain using EⅡB-specific primers CF and CR. (**C**) Identification of ∆EⅡB and C∆EⅡB strains by real-time PCR. Values represent the mean ± SEM (*n* = 3), ** *p* < 0.01.

The deletion strain ΔEⅡB was cultured at 37 °C for 25 generations. Every five generations, the ΔEⅡB strain was identified by D-F and D-R primers (Figure 2A and Supplementary Figure S1A). Stable inheritance of ΔEⅡB was confirmed by amplification of a 952 bp fragment from the deletion strain. Additionally, a band with a size of 376 bp was amplified from CΔEⅡB using C-F and C-R primers (Figure 2B and Supplementary Figure S1B). Sequencing analysis verified the PCR products of ΔEⅡB and CΔEⅡB, while RT-Qpcr verified *EⅡB* gene expression in Lm928, ΔEⅡB, and CΔEⅡB strains (Figure 2C). The results demonstrated that *EⅡB* gene expression was absent in ΔEⅡB strain, whereas it was significantly elevated in CΔEⅡB compared to the wild-type strain (*p* < 0.001) (Figure 2C). These findings confirm the successful construction of both ΔEⅡB and CΔEⅡB strains.

### 3.2. Deletion of EⅡB Did Not Affect Bacterial Growth In Vitro

**Figure 3:**
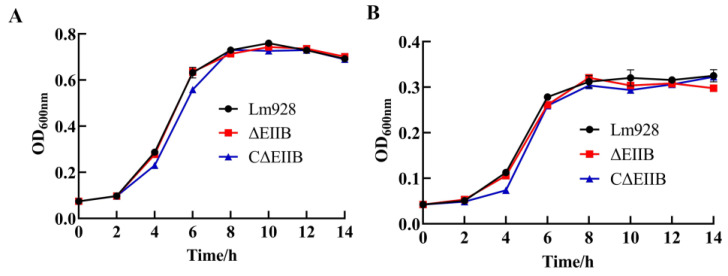
Bacterial growth rates at 37 °C. (**A**) Growth of Lm928, ∆EⅡB, and C∆EⅡB strains incubated in BHI medium; (**B**) growth of wild-type Lm928, ∆EⅡB, and C∆EⅡB strains incubated in cellobiose as the sole carbon source medium. Data presented here are the average of three independent experiments performed in triplicate.

The growth patterns of Lm928, ΔEⅡB, and CΔEⅡB in BHI medium with cellobiose as sole carbon source were consistent (Figure 3). All three strains exhibited an initial adaptation phase from 0 to 2 h, followed by a rapid growth phase from 2 to 8 h, and a final stable growth phase from 8 to 14 h (*p >* 0.05). Compared with the control group, Lm928, ΔEⅡB, and CΔEⅡB strains demonstrated similar glucose, cellobiose, fructose, rhamnose, aesculin, and salicin fermenting abilities when selected as single colonies in the fermentation tube (Supplementary Table S5).

### 3.3. Biofilm Formation Is Defective in ΔEⅡB

**Figure 4:**
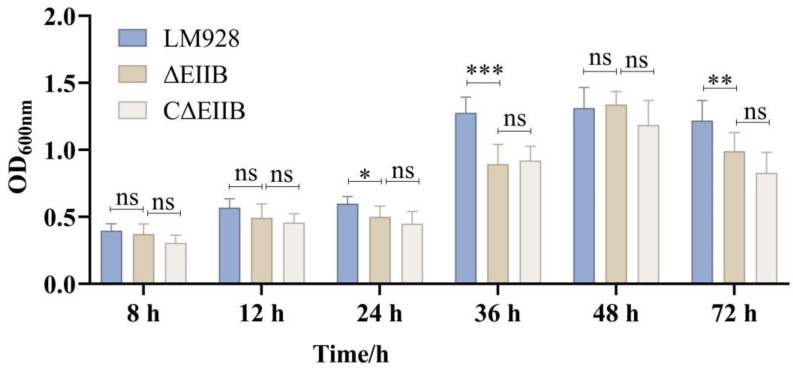
Deletion of EⅡB reduced the ability of *L. monocytogenes* to form biofilms. Biofilm formation for the three *L. monocytogenes* strains was assessed by measuring OD_600_. Error bars represent the standard errors of three biological replicates. Values represent the mean ± SEM (*n* = 6). ns: no significance; * *p* < 0.05, ** *p* < 0.01, *** *p* < 0.001.

The biofilm formation ability of *L. monocytogenes* strains Lm928, ΔEⅡB, and CΔEⅡB was assessed using crystal violet staining. No significant difference in biofilm formation ability was found among these three *L. monocytogenes* strains at 8 h, 12 h, and 48 h (*p* > 0.05). However, compared to the Lm928 strain, ΔEⅡB exhibited significantly reduced biofilm formation at 24 h, 36 h, and 72 h (*p* < 0.05). In addition, there was no significant difference between ΔEⅡB and CΔEⅡB (*p* > 0.05) regarding their amount of biofilm formed (Figure 4).

### 3.4. The Infective Capacity of the Deletion Mutant ΔEⅡB Was Decreased

**Figure 5:**
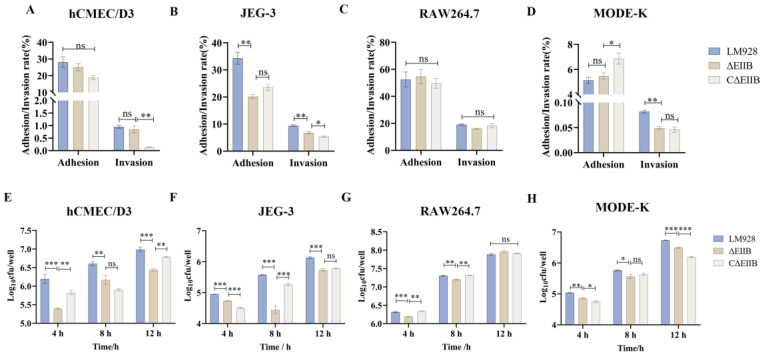
Comparative adhesion, invasion, and intracellular proliferation capacities of Lm928, ΔEⅡB, and CΔEⅡB strains. Three strains infected hCMEC/D3, JEG-3, RAW264.7, and MODE-K cells. After 1 h of infection, the adhesion rate was calculated as the number of cell-associated bacteria, the invasion rate was calculated by adding 100 μg/mL gentamicin-containing medium for 1 h of incubation (recorded as 0 h) (**A**–**D**), the intracellular bacteria number was calculated by adding 10 μg/mL gentamicin-containing medium, and samples were collected at 4, 8, and 12 h, respectively (**E**–**H**). Values represent the mean ± SEM (*n* = 3). ns: no significance; * *p* < 0.05, ** *p* < 0.01, *** *p* < 0.001.

The four cell lines (hCMEC/D3, MODE-K, RAW264.7, and JEG-3) were infected with Lm928, ΔEⅡB, and CΔEⅡB strains and adhesion and invasion assays were conducted. The results demonstrated that compared to Lm928, the adhesion of ΔEⅡB to hCMEC/D3, MODE-K, and RAW264.7 cells did not show significant differences (*p* > 0.05). However, the adhesion ability of ΔEⅡB to JEG-3 was significantly reduced (*p* < 0.01). After treatment with ΔEⅡB, no notable differences in invasion ability were observed between hCMEC/D3 and RAW264.7 cells (*p* > 0.05). In contrast, the invasion abilities of JEG-3 and MODE-K were significantly reduced (*p* < 0.01) (Figure 5A–D).

Four cell lines were infected with Lm928, ΔEⅡB, and CΔEⅡB strains for an intracellular proliferation assay. The results demonstrated that in hCMEC/D3 cells, the amount of intracellular proliferative bacteria was significantly reduced at 4, 8, and 12 h in the ΔEⅡB group compared to Lm928 (*p* < 0.01). Compared to CΔEⅡB, the ΔEⅡB group exhibited a significantly decreased intracellular bacterial count at 4 and 12 h (*p* < 0.01), while no significant difference was observed at 8 h (*p* > 0.05). In JEG-3 cells, a significant reduction in intracellular bacterial count was observed at 4, 8, and 12 h in the ΔEⅡB group compared to Lm928 (*p* < 0.001). Similarly, compared to the CΔEⅡB group, the ΔEⅡB group showed a significantly decreased intracellular bacterial count at both 4 and 8 h (*p* < 0.001), but no significant difference was found at 12 h (*p* > 0.05). In RAW264.7 cells, a notable decrease in intracellular bacterial load was observed at both 4 and 8 h for the ΔEⅡB group compared to Lm928 and CΔEⅡB groups (*p* < 0.01); however, at 12 h, there was no statistically significant difference in the number of intracellular proliferations of the three *L. monocytogenes* strains (*p* > 0.05). In MODE-K, compared with Lm928, the number of intracellular bacteria in ΔEⅡB group decreased significantly at 4, 8, and 12 h (*p* < 0.05) (Figure 5E–H).

### 3.5. Absence of EⅡB Did Not Affect the Hemolytic Capacity of L. monocytogenes

**Figure 6:**
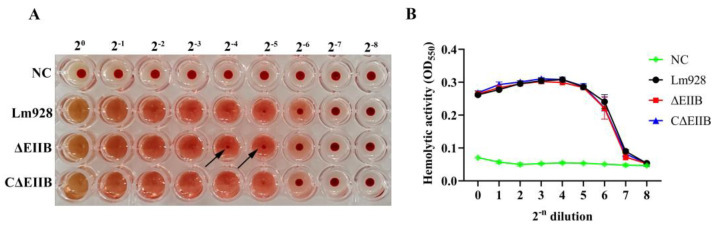
Determination of hemolysis ability of various strains in vitro. *L. monocytogenes* was cultured at 37 °C for 16 h by shaking, and the supernatant was after centrifugation, and the OD_600_ was adjusted to the same value with PBS buffer, then added to a 96-well plate. After double dilution with PBS buffer, an equal volume of 1% sheep red blood cell suspension was added to the plate and BHI medium was used as negative control, incubated at 37 °C for 2 h; after taking photographs (**A**), 100 μL supernatant was aspirated and the OD_550_ value was determined (**B**).

The hemolytic activities of Lm928, ΔEⅡB, and CΔEⅡB were assessed. The results showed that compared with the negative control group, all three *L. monocytogenes* strains were hemolytic. As indicated by the arrow in Figure 6A, the supernatant of group ΔEⅡB was diluted to 2^−4^, and several red blood cells had not ruptured but had instead settled at the bottom, while the red blood cells of groups Lm928 and CΔEⅡB were all ruptured. When diluted to 2^−5^, fewer red blood cells were ruptured in group ΔEⅡB than in the other two groups. However, according to the OD_550_ value, no significant difference in the hemolysis ability of the three *L. monocytogenes* strains was found in vitro (Figure 6B).

### 3.6. Inactivation of EⅡB Impairs the Virulence of L. monocytogenes

**Figure 7:**
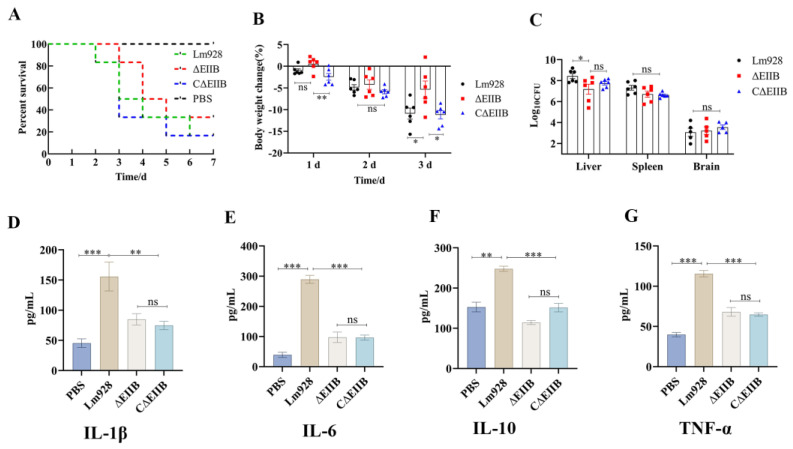
Deletion of EⅡB reduced *L. monocytogenes* virulence and cytokine secretion in mice. Mice were infected with Lm928, ΔEⅡB, and CΔEⅡB strains; (**A**) each mouse was injected intraperitoneally with 10^7^ CFU (group = 6), and deaths were recorded daily for 10 days; (**B**,**C**) each mouse was injected intraperitoneally with 10^5^ CFU (group = 6), weighed on day 0, 1, 2, and 3, and blood was collected on the third. Mice were dissected to obtain liver, spleen, and brain tissues, which were ground, diluted, and plated on BHI agar medium and then counted after 24 h incubation at 37 °C. (**D**–**G**) Detection of IL-1β, IL-6, IL-10, and TNF-α expression levels in mouse serum according to ELISA kit instructions. Values represent the mean ± SEM (*n* = 6). ns: no significance; * *p* < 0.05, ** *p* < 0.01, *** *p* < 0.001.

To assess the impact of EⅡB in LIPI-4 on the virulence of *L. monocytogenes* in mice, 2 × 10^7^ of *L. monocytogenes* solution was intraperitoneally injected into each mouse. Therefore, female mice were chosen in this study. The mice were continuously observed and assessed for a period of 10 days. Following Lm928 infection, mouse mortality began on the second day and peaked between 2–4 d post-challenge. No further mortality was observed after seven days of infection. Mice infected with ΔEⅡB showed mortality on the third day, peaking between the third and fifth day, with no additional deaths occurring from the sixth day onwards. Similarly, mice infected with CΔEⅡB showed mortality on the third day after infection and reached peak mortality between the third and sixth day, but no further deaths occurred after seven days. The average survival times of mice in the Lm928, ΔEⅡB, and CΔEⅡB groups were 3.5 d, 4.5 d, and 3 d, respectively; the survival time of the ΔEⅡB group was 1 d and 1.5 d longer than the survival times of the Lm928 and CΔEⅡB groups, respectively (Figure 7A).

C57BL/6 mice were divided into three groups and intraperitoneally injected with bacterial suspensions of Lm928, ΔEⅡB, and CΔEⅡB. The mice were weighed on days 0, 1, 2, and 3. On the third day, the liver, spleen, and brain tissues of mice were homogenized, and bacterial cells were counted. The results demonstrated that on day 3 (*p* < 0.05), ΔEⅡB mice exhibited significantly less weight loss compared to Lm928 mice (Figure 7B). Furthermore, when compared to mice of the CΔEⅡB group, the body weights of mice in the ΔEⅡB group were significantly lower on both 1 d and 3 d (*p* < 0.05). Regarding bacterial load in the liver tissue, the ΔEⅡB group displayed a significantly lower load compared to the Lm928 group (*p* < 0.05). While a slightly lower bacterial load was observed in the spleen tissue of the ΔEⅡB group compared to the Lm928 group, this difference was not statistically significant (*p* > 0.05). No significant difference in the bacterial load of brain tissue was observed among the three groups (*p* > 0.05) (Figure 7C).

### 3.7. Deletion of EⅡB Decreased the Secretion of Cytokines in Mice

The secretion levels of the cytokines TNF-α, IL-1β, IL-6, and IL-10 in the serum of mice were quantified to gain a deeper understanding of the mechanism underlying the reduced pathogenicity of *L. monocytogenes* in response to *EⅡB* knockout. The results depicted in Figure 7D–G demonstrate that the levels of the four cytokines were lower in mice from the PBS group compared to *L. monocytogenes*-infected mice. Moreover, a significantly higher secretion of cytokines was observed in the Lm928 group compared to both ΔEⅡB and CΔEⅡB groups (*p* < 0.01).

### 3.8. Deletion of EⅡB Affected the Transcription Levels of Virulence and Biofilm-Related Genes

**Figure 8:**
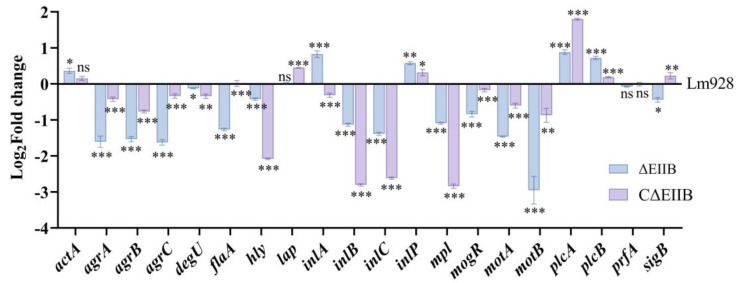
Real-time PCR assays. qRT-PCR was performed on cDNA templates from Lm928, ΔEⅡB, and CΔEⅡB strains harvested 12-14 h after incubation to determine the fold change of virulence and biofilm formation genes. Values represent the mean ± SEM (*n* = 3). ns: no significance; * *p* < 0.05, ** *p* < 0.01, *** *p* < 0.001.

The RNA quality was assessed using agarose gel electrophoresis (Supplementary Figure S2A), and PCR was performed to ensure templates without gDNA (Supplementary Figure S2B). Twenty genes related to virulence and biofilm formation were selected for real-time PCR to determine the gene expression levels of deletion mutants under stationary-phase conditions. The results showed that the transcription levels of *agrA*, *agrB*, *agrC*, *degU*, *flaA*, *hly*, *inlB*, *inlC*, *mpl*, *mogR*, *motA*, *motB*, and *sigB* genes were significantly decreased in ΔEⅡB (*p* < 0.05), while the transcription levels of *actA*, *inlA*, *inlP*, *plcA*, and *plcB* genes were upregulated (*p* < 0.05); however, no significant difference was found in the mRNA levels of *lap* and *prfA* (*p* > 0.05) (Figure 8).

## 4. Discussion

In recent years, the frequency of the occurrence of food poisoning caused by *L. monocytogenes*-contaminated food has increased. This increased occurrence has elevated listeriosis as a key foodborne infectious disease with detrimental effects on global food safety and human health [27]. To survive and reproduce, the intracellular parasite *L. monocytogenes* depends on the acquisition of nutrients from host cells, which ultimately harms host animals. The utilization of carbon sources plays a crucial role in the survival and pathogenicity of *L. monocytogenes*. Analysis of the entire sequence of the *L. monocytogenes* type strain EGD-e showed that *L. monocytogenes* has a total of 86 PTS genes. These genes encode 29 complete PTSs that are responsible for the transport of carbohydrates and sugar alcohols [28]. Additionally, several single PTS components were found that may also aid in the transport of these compounds [28]. The experiment showed that removal of *EⅡB* did not impact the growth curve and sugar fermentation (Figure 3, Supplementary Table S5), aligning with the results of a previous study [15]. These findings indicate that EⅡB does not play a role in the mechanism of *L. monocytogenes* proliferation in vitro, possibly because other PTS components in *L. monocytogenes* are responsible for the transport of cellobiose.

Specific PTS systems have been associated with bacterial virulence and biofilm formation. In *Salmonella*, transcriptome analysis revealed decreased expression of genes involved in quorum sensing ability, *Salmonella* pathogenicity island, flagella, and PhoPQ regulon in PTS mutant [29]. Research even showed that in *Salmonella*, *pts* I and *crr* deletions (components of the PTS) have the potential to provide an effective attenuated mucosal live vaccine [30]. In *Bacillus anthracis*, AtxA transcription, which encodes the major virulence gene regulator *atxA*, was decreased in both HPr and EI deletion and phosphoric acid transfer mutants. These changes affected the production of anthrax toxin, and the virulence of HPR-EI mutants was reduced in an anthrax mouse model [31]. In *Streptococcus mutans,* research demonstrated that the absence of the asc-PTS system reduced the growth rate and biofilm-forming capacity [12]. In *Cronobacter, malX* gene (encoding maltose transporter subunit ⅡCB) knockout resulted in decreased extracellular polysaccharide content and decreased cellulose-related gene expression, thereby also reducing biofilm formation [16]. In *L. monocytogenes,* research showed that deletion of *EⅡB* (LmOf2365-0443) significantly reduced the spread ability of F2365 strain in HT-29 cells [17]. In addition, deletion of *EⅡB^man^* enhanced the motility, hemolytic activity, and proliferation in RAW264.7 cells, as well as bacterial load in mouse tissues of the EDG-e strain [15]. In contrast, the experiment conducted in this study showed that deletion of EⅡB resulted in deficiencies in biofilm formation and also decreased the intracellular proliferation ability of Lm928 in different cell lines The deletion of *EⅡB* decreased the virulence of the Lm928 strain, as indicated by increased survival times of mice, decreased bacterial load in liver tissue, and reduced degree of weight loss in the ΔEⅡB group (Figure 7A–C). These findings align with the function of the EⅡB protein in the cellobiose phosphotransferase system (cel-PTS) of *Streptococcus agalactis* [32]. A significant decrease in biofilm formation ability and tilapia mortality has been identified by cel-EⅡB deletion in *Streptococcus agalactiae* strain THN0901. The virulence gene mRNA expression of *csrS*, *csrR*, *rgfA*, *rgfC*, *bgrR*, and *bgrS* was considerably reduced in the Δcel-EⅡB strain [32]. *Streptococcus mutans* JAM1 strain lacking *E*Ⅱ*AB^Man^* also had lower expression levels of exopolysaccharide-forming glucosyltransferases, indicating an involvement in the regulation of sucrose-dependent biofilm formation and virulence [33]. Further, research showed that removal of the *manL* gene, which encodes the Man-EⅡAB protein, led to changes in at least 10 functional gene expression categories [28]. Therefore, this study examined whether the reduced virulence of Lm928 resulting from *EⅡB* deletion was connected to its control of other crucial virulence genes.

The authors postulate that the decreased virulence of Lm928 resulting from *EⅡB* deletion is linked to the control of genes related to virulence [32,34]. Therefore, the transcript levels of 20 genes associated with virulence and biofilm formation were analyzed in the deletion strain. Among these genes, 65.0% (13/20) exhibited downregulation, including *agrA*, *agrB*, *agrC*, *degU*, *flaA*, *hly*, *inlB*, *inlC*, *mpl*, *mogR*, *motA*, *motB*, and *sigB* genes. Furthermore, expressions of a quarter (25.0%) of genes, specifically *actA*, *inlA*, *inlP*, *plcA*, and *plcB*, had increased. No significant changes were observed in the *prfA* and *lap* genes (Figure 8). The ability of *L. monocytogenes* to adhere to, invade, reproduce, and spread in various human tissues and organs depends on numerous virulence factors. Essential virulence genes are mainly concentrated in *L. monocytogenes* LIPI-1 (including *prfA*, *plcA*, *hly*, *mpl*, *actA*, and *plcB*) and LIPI-2 (such as *inlA*, *inlB*, and *inlC*), the expression of which is regulated by PrfA [35,36]. This study found no significant change in the transcription level of *prfA*, and the protein activity of PrfA was controlled by culture conditions [37]. PrfA protein activity was reduced in *L. monocytogenes* in a nutrient-rich medium [37]. However, this study found that certain genes regulated by PrfA were upregulated, while others were downregulated. It can be theorized that these genes could likewise be controlled by SigB, which is synthesized by the *sigB* gene and serves as the primary regulator of the response to environmental stress [38]. The genes *bsh*, *inlA*, and *inlB* are experimentally verified SigB-dependent virulence genes [39]. A study showed that the expression levels of *inlA* and *inlB* genes in EGDe were remarkably downregulated after *sigB* deletion, while virulence genes such as *act A*, *plcA*, and *plcB* were upregulated 4.5-fold [27]. In the present study, *plcA* and *plcB* transcripts were upregulated under *sigB* downregulation, while *inlB* transcripts were significantly downregulated, which is consistent with the findings of previous research [27].

In the present study, the transcript levels of *agrA*, *agrB*, *agrC*, *degU*, *flaA, mogR, motA*, and *motB* decreased in the deletion strain. Previous research showed that these genes are related to the virulence and biofilm formation of *L. monocytogenes* [40,41,42,43,44,45,46]. Researchers have identified two quorum sensing systems in *L. monocytogenes*: the Lux S system [47] and the Agr system [42]. The *agr* locus of *L. monocytogenes* consists of an operon containing *agrBDCA*. Research has indicated that the Agr system influences the virulence of *L. monocytogenes* and its biofilm formation ability [40,41,42]. The flagella subunit of *L. monocytogenes* is encoded by the *flaA* gene. After a frameshift mutation of the *flaA* gene, the Xen32 strain of *L. monocytogenes* showed defective flagella formation and weakened virulence in mice [43]. MotA and MotB are two dynamic proteins present on the surface of bacterial cells that generate the energy required for flagella movement. Research has shown that *L. monocytogenes* Δ*motA* mutation reduces the surface attachment of cells, thus affecting the initial adhesion stage of biofilm formation [44]. Compared with the wild-type strain, the Δ*flaA* and Δ*motAB* mutant strains showed reduced biofilm formation and significantly smaller colony diameters on semisolid agar [45]. DegU is involved in the motility, chemotaxis, and biofilm formation of *L. monocytogenes* [46]. After the *degS* genes of *B. subtilis* were introduced into *L. monocytogenes* without DegS, the expression of DegU in *L. monocytogenes* strains increased, and both the motility and chemotaxis of *L. monocytogenes* were enhanced [46]. The motility and chemotaxis of *L. monocytogenes* decreased in the *degU* deletion group, which also impacted biofilm formation [46]. Overall, this study found that the deletion of *EⅡB* led to the downregulation of major virulence genes, resulting in lower virulence and biofilm formation capabilities in the deletion strains.

Monk et al. (2008) developed an integrated *L. monocytogenes* vector named pIMK2, which includes the potent promoter pHelp to enhance gene expression [48]. pIMK2 has been extensively utilized to create *L. monocytogenes* complement strains [23,26,48]. In the present study, potent promoter complement strains were built. The expression of EⅡB in CΔEⅡB was significantly higher than in Lm928, presenting a 110-fold increase (Figure 2). CΔEⅡB functioned as an EⅡB overexpression strain. However, in subsequent tests, most biological traits of CΔEⅡB did not return to the level of the wild-type strain. When compared to ΔEⅡB strains, CΔEⅡB strains exhibited similar biofilm formation ability, bacterial load, and virulence gene transcription levels. Similar findings were reported by Cheng et al. (2021) [26]; after overexpression of the *yjbH* gene in the complemented strain, the biological characteristics of the strain were closer to those of the deletion strain. A prior study put forward the following suggestion: independent of whether gene complementation is achieved through plasmid or insertion vector, the natural state is not precisely replicated because of variations in the genetic machinery and topology of the complemented gene [49]. The authors of the present study agree with the above, namely, that expression of genes in the complemented strain does not restore the strain to the level of the wild-type strain.

Following *Listeria* infection in animals, the levels of immune-related cytokines will change, thus prompting the body to generate humoral and cellular immunological responses. In this study, the levels of IL-1β, IL-6, IL-10, and TNF-α in the sera of mice were measured three days after infection with Lm928, ΔEⅡB, and CΔEⅡB. The results indicated that EⅡB stimulated the generation of pro-inflammatory cytokines IL-1β, IL-6, and TNF-α (Figure 7D–G). These cytokines are essential for initiating Th1 immune responses as effector cytokines. Research has demonstrated that infection of wild-type mice with *L. monocytogenes* leads to elevated levels of blood IL-10 levels 3–4 days after infection [50,51], which aligns with the findings of this study. IL-10 deficient mice eliminate *L. monocytogenes* more quickly than wild-type mice, suggesting that IL-10 plays a role in diminishing the initial innate immune response to *L. monocytogenes* [52,53]. Thus, the decrease in IL-10 secretion by the mice in the deletion group may enhance their bodies’ ability to eliminate *L. monocytogenes*, leading to a drop in tissue bacterial load. Collectively, these findings indicate that EⅡB in *L. monocytogenes* LIPI-4 stimulates the release of inflammatory factors IL-1β, IL-6, IL-10, and TNF-α to combat infection.

## 5. Conclusions

This study found that the biofilm formation (Figure 4) and cell intracellular proliferation (Figure 5E–H) ability of *L. monocytogenes* were defective in response to *EⅡB* knockdown. The virulence of *L. monocytogenes* in mice and cytokine expression levels were also reduced (Figure 7A–G). Transcription levels of 65% (13/20) of genes associated with virulence and biofilm formation were reduced in the mutant strain (Figure 8). It can be speculated that EⅡB is involved in the regulation of these genes, thereby reducing the virulence of *L. monocytogenes* both in vivo and in vitro. This result lays a solid foundation for exploring the role of EⅡB and LIPI-4 in the pathogenesis of *L. monocytogenes*.

## Data Availability

The original contributions presented in this study are included in the article; further inquiries can be directed to the corresponding author.

## Supplementary Materials

The following supporting information can be downloaded at: https://www.mdpi.com/article/10.3390/vetsci11070301/s1, Figure S1: The EⅡB deletion and complement strains were successfully constructed; Figure S2: Detection of RNA quality and the template was checked for the presence of gDNA. Table S1: The PCR primers were utilized for the construction and identification of the deletion and complementation strains. All primers were synthesized by Sangon Biotech, China; Table S2: Splicing with overlap extension PCR reaction system; Table S3: SOE-PCR reaction conditions; Table S4: The information of Real-time PCR primers in this study; Table S5: Results of carbohydrate fermentation test.
