# Supplementary material for: EⅡB Mutation Reduces the Pathogenicity of Listeria monocytogenes by Negatively Regulating Biofilm Formation Ability, Infective Capacity, and Virulence Gene Expression
Source: Vet Sci. 2024 Jul 2;11(7):301. doi: 10.3390/vetsci11070301 (PMC11281496; doi:10.3390/vetsci11070301)
Supplement: Supplementary file 1 [file vetsci-11-00301-s001.zip › vetsci-3034449-supplementary.pdf]

Table S1 The PCR primers were utilized for the construction and identification of the deletion and complementation strains. All primers were synthesized by Sangon Biotech, China.

Primers	Sequence（5′ →3′ ）	Restriction site	Amplicon size(bp)
Mutant construction			
Upstream homologous recombination arm primers			
F1	*AA*CTGCAGGCCAGTAATGTTTGGTGTTCC	*Pst* Ⅰ	344
R1	CTGTAATTCAGTGCCTTCCAGTCTATCGTCCT		
Downstream homologous recombination arm primers			
F2	AGGACGATAGACTGGAAGGCACTGAATTACAG		375
R2	*CG*GAATTCTGATTGCTCTTGTGAACCC	*Eco* R Ⅰ	
Detecting primers			
D-F	GTTACAGGAGCACTCGTTGTC		952(1309)
D-R	GAGATGTAAGCCTATTCCAAG		
Complementary construction			
C-F	*AA*CTGCAGGTAGGAAAGGACGATAGACATGAAA	*Pst* Ⅰ	376
C-R	*CCG*CTCGAGTTAGTTTCCCTCCGTCTTAAATGTA	*Xho* Ⅰ	

Note: The protective bases are set to italic; The restriction enzyme sites are underlined.

Table S2 Splicing with overlap extension PCR reaction system.

Reaction steps	Reaction system	Volume of reaction (μL)	Total volume (μL)
One	PCR product purified by the upstream homology	2.0	30.0
	PCR product purified by the downstream homology	2.0	
	Taq Plus Master Mix II	20.0	
	Ultrapure water	6.0	
Two	F1 primers	2.0	35.0
	R2 primers	2.0	
	dNTP (TransGen Biotech, China)	1.0	
	First step product	30.0	

Table S3 SOE-PCR reaction conditions.

Reaction steps	Reaction conditions (℃)	Reaction time (min)	Number of cycles
	95	5.0	1
	95	0.5	
One	58	0.5	18
	72	1.0	
	72	10.0	1
	95	5.0	1
	95	0.5	
Two	58	0.5	35
	72	1.0	
	70	10	1

Table S4 The information of Real-time PCR primers in this study

Name	Forward primer sequence (5′ →3′)	Reverse primer sequence (5′ →3′)	Amplicon size(bp)
actA	CGACATAATATTTGCAGCGAC	CGTGAACTTACTTCACGTGCA	138
agrA	GCAGCCGGACATGAATGG	AACCACGCGGATCAAACTTC	62
agrB	TCGCTCCGGCAGACACA	TTTTTAGTGTTTTCCGGTGTTCTTC	63
agrC	TATTTTGCTAGATAATGCGGTTGAA	CGCGATTCGAATAACTGGATTT	64
degU	ACGCATAGAGAGTGCGAGGTATT	CCCAATTCCGCGGTTACTT	63
EⅡB	TCTCAATGGCTTATGAACAG	GCAGCGATTAGAATTGGTAC	121
flaA	GACTTGTTACAAACAGAGGATTCA	ATTGACGCATACGTTGCAAGAT	67
gyrB	AGACGCTATTGATGCCGATGA	GTATTGCGCGTTGTCTTCGA	91
hly	ATTGCGCAACAAACTGAAGC	TCGATTGGCGTCTTAGGACT	110
lap	TATTATCCGGTGTGACGTGG	TGGTCAAGTCCGGCAAGTG	143
inlA	AAAGATATAGGCACATTGGCGAG	GACCCGACAGTGGTGCTAGATTA	91
inlB	GTGAAAGAAAAGCACAACCCAAG	TCGCCCCGTTTCCAATAATTAT	94
inlC	AAAACCAAGCATCAACAATACT	TGTTTTTAGATAACAACGAACTC	113
inlP	CCAACTTCCGACGAC	CTCCAGTGGCATAATTGT	116
mpl	GGCGTTACGCATTATACGC	TATTATCCGGTGTGACGTGG	88
mogR	AACTGCCGAAGAAATCTACCATTT	CGATTCCACCGTGTTCTTCA	68
motA	CAACGCTCGGTGTACTTGGA	TTTCGCCCATCGCATGA	54
motB	TGCAAAAAAATTCGAACAAATGG	CTGCCGCGCCTTCCT	62
plcA	CAACTAGAAGCAGGAATACGGTAC	TGAGTAATCGTTTCTAATACACCTG	116
plcB	TATCAAGCAACAGAAGACATGGT	TGACTATTTTCGGGTAGTCCG	109
prfA	GCCAACCGATGTTTCTGTATCA	TGGTATCACAAAGCTCACGAGT	115
sigB	GTTGGCGAAATTAAACGA	CGGCGAGCTTTGTAGTTC	129

Table S5 Results of carbohydrate fermentation test

Strains	Glucose	Cellobiose	Fructose	Rhamnose	Esculin	Salicin
Lm928	＋	＋	＋	＋	＋	＋
ΔEⅡB	＋	＋	＋	＋	＋	＋
CΔEⅡB	＋	＋	＋	＋	＋	＋

“＋” Positive

A

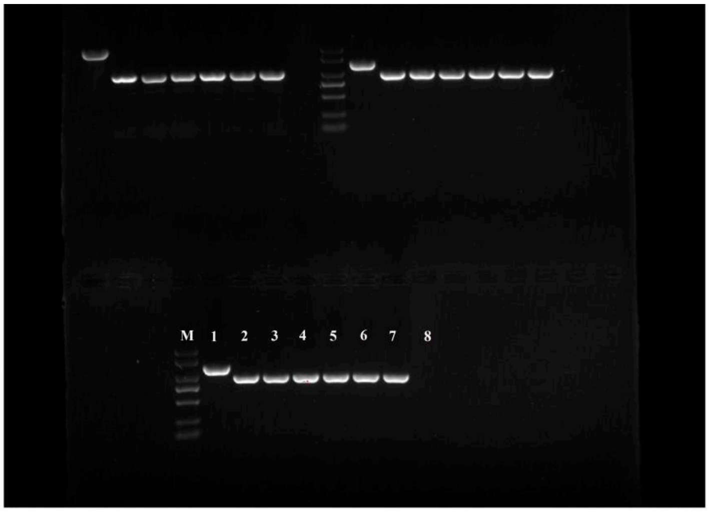

B

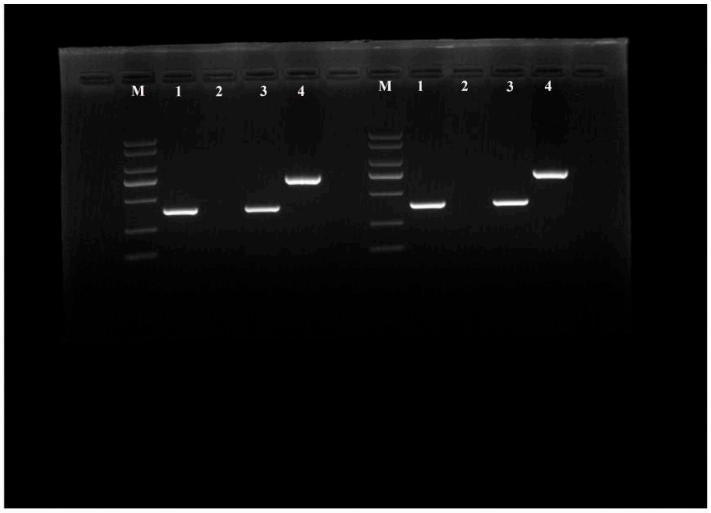

Figure S1. The EⅡB deletion and complement strains were successfully constructed. A, M: DL2000 DNA Marker, 1: The PCR products of strain Lm928 were amplified with DF and DR Primers; 2-7: The PCR products of generation 1, 5, 10, 15, 20, and 25 strains of strain ΔEIIB were amplified with DF and DR Primers; 8: Negative control. B, M: DL2000 DNA Marker. 1-3: PCR products of LM928, ΔEⅡB and CΔEⅡB were amplified with CF and CR primers. 4, PCR product of CΔEⅡB strain amplified by *hly*-F/*hly*-R primer (*hly*-F:CTGAATTCGGCTGTTACTAAAGAGCAGTTGC; *hly*-R:ATGGATCCTTAGCCCCAGATGGAGATATTTCTA. PCR product size was 749 bp).

A

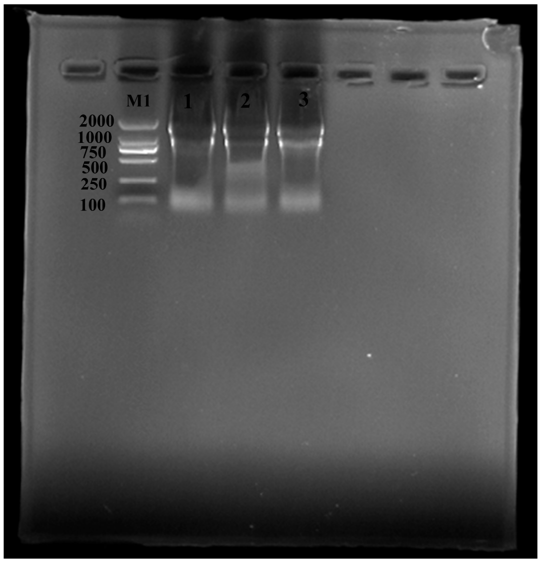

B

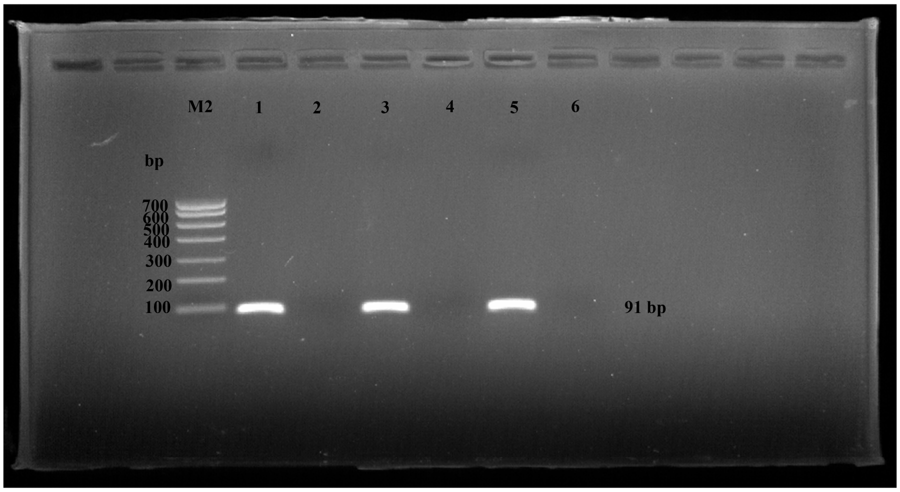

Figure S2. Detection of RNA quality and the template was checked for the presence of gDNA. A, RNA electrophoresis in 0.5×Tris-borate-EDTA (TBE) buffer. M1: Trans2K DNA Marker (TransGen Biotech, China); 1, 2, 3 are total RNA (3 μg) of Lm928, ΔEIIB and CΔEIIB strains, respectively. Total RNA was extracted using an RNA extraction kit, electrophoresis was performed in 1.0% agarose gels containing 0.5× TBE buffer (0.045 M Tris–borate, 1 mM EDTA), which was also used as a running buffer, 50 mL agarose gels were run at 160 V/cm for 20 min. B, The gDNA signal in the template was excluded. M2: Trans DNA Marker I (TransGen Biotech, China); 1, 3, 5: the housekeeping gene *gyrB* was amplified by PCR using the cDNA(RT reaction) of Lm928, ΔEⅡB and CΔEⅡB as templates; 2, 4, 6: the housekeeping gene *gyrB* was amplified by PCR using Lm928, ΔEⅡB and CΔEⅡB negative control cDNA (No RT Control, without reverse transcriptase) as templates. The RT reaction system: Total RNA 1μg, 5×All-in-One Reaction Mix for qPCR 4 μL, *TransScript*® Uni All-in-One Enzyme Mix (or 20×No RT Control Mix) 1 μL, RNase- free Water was added to a total volume of 20 μL; The RT reaction condition: 50 ℃ for 5 min, followed by 85 ℃ for 2 min. Following RT, the *gyrB* gene was amplified by PCR using cDNA as template, PCR products were subjected to 1% agarose gel electrophoresis to detect the presence of gDNA in the template.
